# A “silent storm”: uncovering the escalating crisis in mental healthcare for children and adolescents in Slovenia during and after the COVID-19 pandemic

**DOI:** 10.1186/s13034-024-00811-2

**Published:** 2024-11-05

**Authors:** Sanja Zupanič Mali, Sašo Karakatič, Maja Drobnič Radobuljac

**Affiliations:** 1 Faculty of Medicine, University of Ljubljana, Vrazov trg 2, 1000 Ljubljana, Slovenia; 2 Unit for Intensive Child and Adolescent Psychiatry, University Psychiatric Clinic Ljubljana, Center for Mental Health, Grablovičeva 44b, 1000 Ljubljana, Slovenia; 3 Department of Psychology, Developmental Psychopathology Lab, Health and Biomedical Sciences Building, University of Houston, 4811 Calhoun Rd, Houston, TX 77204-6022 US; 4 Faculty of Electrical Engineering and Computer Science, Laboratory of Intelligent Systems, University of Maribor, Koroška cesta 46, 2000 Maribor, Slovenia

**Keywords:** Child and adolescent mental health, Healthcare system burden, Waiting times, COVID-19

## Abstract

**Aim:**

Our aim was to assess the burden of children and adolescents’ mental health problems on the Slovenian outpatient healthcare system before, during and after the pandemic.

**Methods:**

In a retrospective analysis of healthcare indicators from 2008 to 2023, we analysed data from the National Institute of Public Health. Key domains included initial visits for mental and behavioural disorders (MBDs) to primary care for the population aged 0–19; the number of referrals to an initial assessment with a child and adolescent psychiatrist (CAP) at the secondary level for the population aged 0–17 along with the corresponding waiting times; and the number of urgent referrals for the population aged 0–17 to emergency mental health centres at the tertiary level. The calculations included rates per 1000 people. Descriptive statistics and diagrams were used to compare the data. Segmented linear regression analysis (SLR) was conducted on the primary healthcare data to identify the distinct temporal point indicating an increase.

**Results:**

Comparing the average rates of the 2020–2022 period to those of the 2018–2019 period, there was a 20% increase in initial visits to primary care, a 23% increase in the referral rate to a CAP at the secondary level, and a 41% increase to the tertiary level of care. In secondary care, a four- to sevenfold increase in waiting times for the initial CAP assessment was observed between 2019 and 2023. The incidence of initial visits to primary healthcare services for MBD increased from 2008 to 2019 (average annual growth rate of 4.5%). The average annual growth rate for the 2020–2022 period tripled to 13.9%. The SLR showed that the trend of accelerating growth could have begun in 2017 for the 0–5 age group and possibly for the 15–19 age group as well.

**Conclusions:**

After the initial decline in 2020, all levels of the Slovenian healthcare system faced an increased burden of MBD in children and adolescents from 2021 to 2022 compared to pre-pandemic levels. Nevertheless, a portion of this increase aligns with longitudinal growing trends from 2008 onwards. Tackling the crisis requires urgent national action, significant improvement in organization, and investments in mental health.

**Supplementary Information:**

The online version contains supplementary material available at 10.1186/s13034-024-00811-2.

## Background

From January 2020 to May 2023, COVID-19 was declared an international health emergency by the World Health Organization [1]. The COVID-19 pandemic wrought a negative impact on the mental health of children and adolescents worldwide [2–7]. However, there is still no consensus on the extent of this impact [8]. The data from other comparable situations show that the consequences of social isolation and loneliness on young people’s mental health can be long-lasting [9]. In addition, the data suggest that families are still struggling with finances, increased screen time, restricted social leisure activities, an unstable global political situation, and increased levels of violence among children and adolescents, all of which contribute to an ongoing mental health crisis for this demographic [10–13].

Due to the pandemic, Slovenia saw strict restrictions in 2020 and the first half of 2021, with widespread school closures and limited social life. However, as in other European countries, child and adolescent psychiatrists (CAP) quickly switched to telecommunication to stay in contact with their patients, and emergency services in three university hospitals remained accessible [14, 15]. Despite these efforts and the significant impact of mental health treatment on adolescent suicidality [16, 17], there was a statistically significant increase in suicide attempts and suicidal thoughts among children and adolescents in Slovenia in 2020 and the first half of 2021 compared to the corresponding period before the onset of COVID-19 [15].

Even in countries with robust child and adolescent mental health services (CAMHSs), there were disruptions to the provision of mental health services during the pandemic [8, 14, 18]. A typical pattern was an initial decrease in referrals followed by a sharp increase [19, 20]. However, access to child and adolescent mental healthcare in Slovenia was inadequate before the start of the pandemic [21, 22]. An increase in anxiety disorders and antidepressant prescribing in children and adolescents was reported between 2008 and 2015, and there have been increasing efforts to expand mental healthcare for this age group [21]. In 2023, there were approximately 45 active CAP specialists in Slovenia, compared to 25 in 2016 [23].

Slovenia has a three-tier public healthcare system. For children and adolescents with mental and behavioural disorders (MBDs), the primary level provides access to paediatric services, family medicine, and emergency services. At this level, patients are referred to secondary care, where CAPs work in CAMHS centres (CAMHC) or concession outpatient clinics. In Slovenia, referrals are categorized based on urgency into ‘very fast’ (where patients should receive their initial appointment with a CAP within 14 days), ‘fast’ (within 3 months), and ‘regular’ (within 6 months)– all of these referred patients are then assessed at the secondary level. Emergencies, such as those with suicide risk or severe psychiatric problems, are assessed and treated in one of the 3 triage emergency centres located at tertiary university hospitals. The latter are referred to with an ‘urgent’ designation and require assessment within 24 h [21, 22].

Our aim was to assess the burden of mental health problems on children and adolescents in the Slovenian outpatient healthcare system before, during and after the COVID-19 pandemic, and to compare the data with those from the pre-pandemic period.

## Methods

We conducted a retrospective analysis of healthcare indicators for the period from 2008 to 2023, covering the age group of 0–19 years.

First, we collected the statistical data from the National Institute of Public Health (NIPH), and all relevant material is included in Appendices 1 and 2. The data covered the following key areas:

- The number of initial visits to primary healthcare services related to MBD (data were accessible from 2008 to 2022). These services are integrated into the public health system and are required to submit data to the NIPH database as mandated by the Health Care Data Collection Act [24] (see Appendix 1). As a result, minimal data loss occurs. Diagnoses are coded according to the International Classification of Diseases, 10th Revision (ICD-10), in the category of “Mental and behavioural disorders” (F00–F99). A specific initial diagnosis can be reported only once within a calendar year.
- The number of regular, fast, and very fast referrals for initial assessment with a CAP (data available from 2018 to 2022) is an important indicator of the strain on the secondary care system.
- The average waiting times for initial assessment with a CAP (data available from 2018 to 2023) as of August 21. This date was chosen at random. Waiting times are given in days and show the efficiency of the secondary care system in answering the demands.
- The number of urgent referrals for initial assessment with a CAP (data available from 2018 to 2022). The load on tertiary emergency centres can be measured by the number of urgent referrals.

Second, using the data on the number of residents within each year and age group [25], we calculated the following:

- The rate of initial visits to primary healthcare services related to MBD per 1000 individuals aged 0–19 years.
- The rate of referrals for initial assessment with a CAP per 1000 individuals aged 0–17 years. Notably, for adolescents with mental health problems in the transition to adulthood at the age of 18, the Slovenian Health Insurance Institute no longer covers the cost of the initial CAP assessment. Consequently, these individuals are referred to psychiatrists. It is important to emphasize that some patients with an urgent referral did not visit the emergency centre at all. Conversely, it is noteworthy that one of the three emergency centres, the only one operating 24/7, also accommodates patients without a referral.

We compared the rate data from the pre-pandemic years of 2018–2019 to the data from the 2020–2022 period for all three levels of the healthcare system. After graphically presenting the primary care service data, we calculated the average annual growth rate of initial visits to primary healthcare services related to MBD per 1000 individuals aged 0–19 years for two distinct periods: the pre-pandemic years (2008–2019) and the pandemic period (2020–2022). The annual growth rate for each year was determined by comparing the rate of that year to the rate of the previous year using the following formula: (Year(X) rate– Year(X-1) rate)/Year(X-1) rate * 100. Afterwards, we tried to determine when the acceleration of growing trends for the rate of first visits to primary care for MBDs per 1000 people began. We conducted a segmented regression analysis on the data concerning the rates of initial visits for MBD per 1000 individuals for each age group separately and for all age groups combined. As the number of visits showed an anomaly in the first year of the COVID-19 epidemic, two segmented linear regression models were built– one with 2020 included and one without. The analysis was conducted using R version 4.4.0, with the ‘nlme’ and ‘segmented’ packages to fit and segment the linear models.

Third, because of some doubts in the media about the reliability of NIPH data on waiting times, we initiated a cross-sectional study to collect data on waiting times directly from secondary-level services. We contacted all 23 CAP providers in Slovenia by telephone in the second half of August 2022. After introducing the study, we presented a fictitious case of a 16-year-old female high school student who received a very fast referral at the emergency assessment after attempting suicide. We collected the data available for the initial assessment from all the providers. The response rate was 91% (more details are provided in Appendix 3). The results were then compared with the official NIPH data from 21 August 2022, confirming the reliability of the NIPH data on waiting times. Finally, in August 2022, we also interviewed the heads of all three tertiary CAP hospital departments. These tertiary hospitals serve as primary assessment centres for all psychiatric emergencies in the under-18 population. The main objective of these interviews was to gain insight into their strategies for managing the increasing influx of emergency patients (Appendix 3).

## Results

### Primary healthcare

Comparing the pre-pandemic years 2018–2019 with the period 2020–2022, the rates of initial visits to primary healthcare services for MBD in the 0–19 age group increased from 22.59 per 1000 people to 27.17 per 1000 people, indicating a 20% increase, despite a 12% decrease in visits in 2020 compared to 2018–2019 (see Appendix 1). Nevertheless, the incidence of initial visits to primary healthcare services for MBD in the 0–19 age group has risen steadily since 2008, with an average annual growth rate of 4.5% observed between 2008 and 2019. In the pandemic year 2020, a significant decrease of 18.7% in these initial consultations was observed compared to the 2019 figures. The increase in visits in the following two years had an average annual growth rate of 13.9% for the period 2020–2022, reflecting a 3.1-fold increase in the growth rate compared to that in the years before the pandemic.

**Fig. 1:**
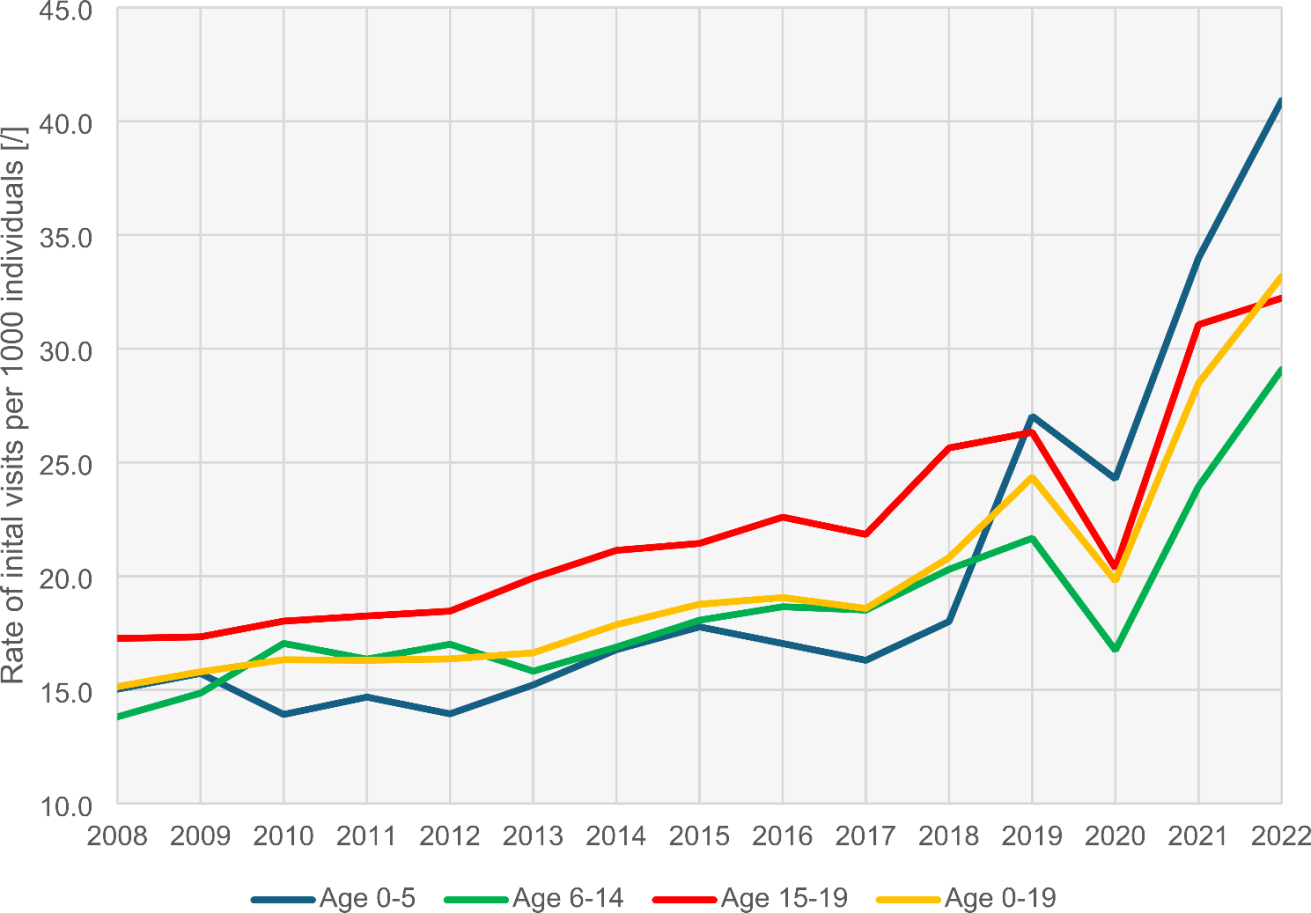
The rate of initial visits to primary healthcare services for mental and behavioural disorders per 1000 individuals aged 0–19 years

A closer look at the age-stratified data revealed similar trends in all age groups (see Fig. 1). In 2022, the rate of initial visits to primary healthcare services for MBDs was 40.9 per 1000 children aged 0–5 years, compared to 27.0 in 2019 and 15.0 in 2008. In the 6–14 age group, the rate was 29.1 per 1000 children in 2022, compared to 21.7 in 2019 and 13.8 in 2008. For adolescents aged 15–19 years, there were 32.2 initial consultations related to MBD per 1000 adolescents in 2022, compared to 26.3 in 2019 and 17.3 in 2008. For children and adolescents aged 0–19 years, the rate increased from 15.1 per 1000 in 2008 to 24.4 in 2019 and further to 33.2 in 2022.

**Fig. 2:**
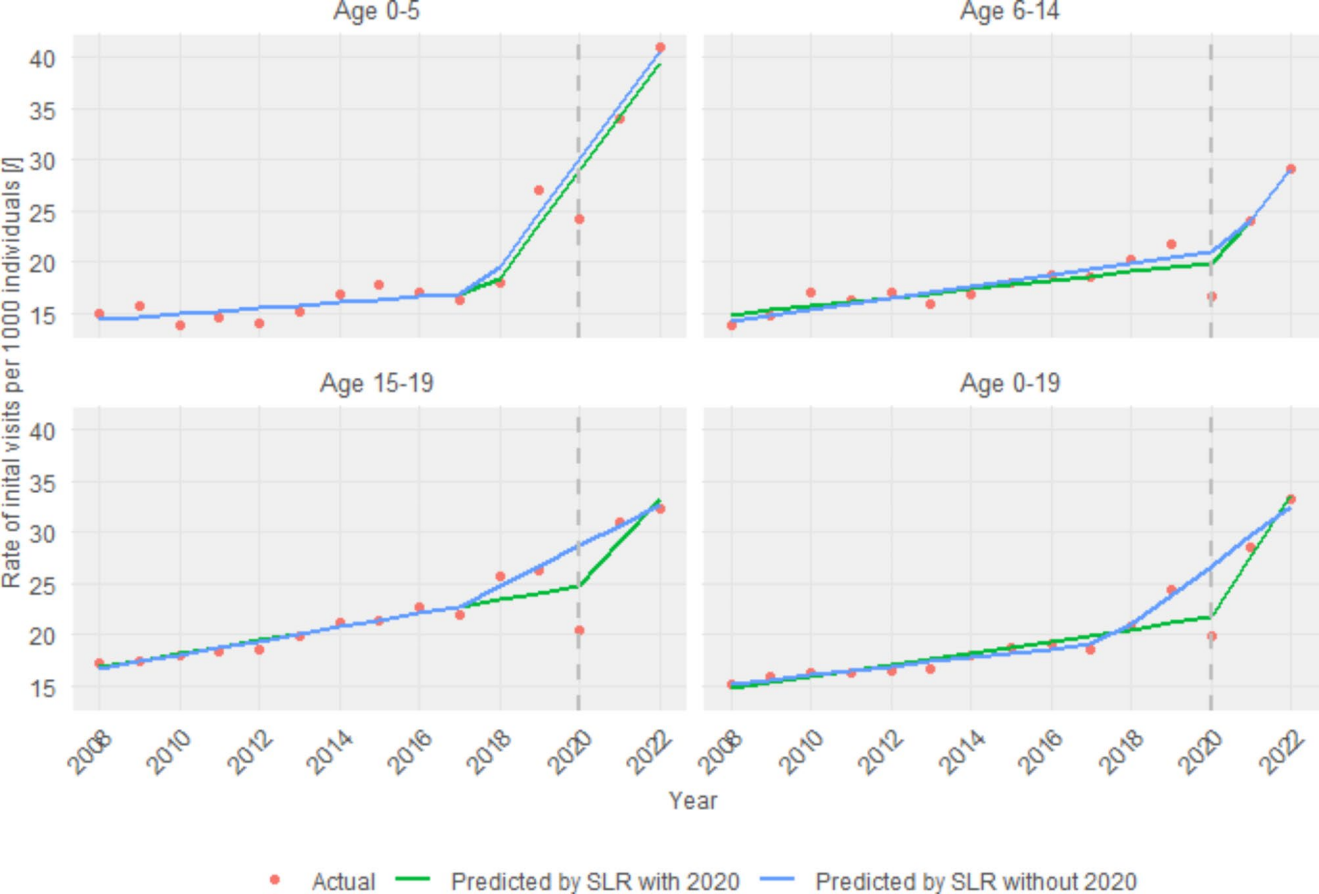
Segmented regression analysis on the data concerning rates of initial visits at primary healthcare for mental health issues per 1000 individuals for all age groups separately and all together. *SLR* segmented linear regression

In an exhaustive examination of segmented linear regression models, interesting patterns emerge when comparing models inclusive and exclusive of the anomalous year 2020 (see Fig. 2).

For children aged 0–5, the model incorporating 2020 identified a breakpoint in the second half of 2017, exactly in 2017.79 (SE = 0.43), with a significant change in the post-breakpoint slope (β = 4.99, SE = 0.67, t = 7.46). This finding is in line with the model excluding 2020, which similarly identified a breakpoint in 2017.56 (SE = 0.30) with a similar post-breakpoint slope (β = 4.99, SE = 0.49, t = 11.13). Both models exhibited high adjusted R^2^ values, indicating excellent model fits (0.94 and 0.97, respectively); however, the exclusion of 2020 led to a slightly tighter fit.

For the 6–14 age group, the model with 2020 pinpointed a breakpoint in 2020.23 (SE = 0.59), showing a less pronounced post-breakpoint slope change (β = 4.72, SE = 1.98, t = 2.39) compared to the model without 2020, which chose 2020.50 (SE = 0.33) as the breakpoint and demonstrated a slightly sharper slope increase (β = 4.58, SE = 1.25, t = 3.66). This age group showed a more substantial decline in model fit when 2020 data were included (adjusted R^2^: 0.87 vs. 0.95).

The 15–19 age group models revealed that including data from 2020 identified the expected breakpoint at 2020 (SE = 1.17), with a moderate increase in the post-breakpoint slope (β = 3.61, SE = 2.61, t = 1.38). When 2020 was omitted, the breakpoint was computed for a much earlier year, 2017 (SE = 0.59), and the post-breakpoint slope increase was significantly lower (β = 1.31, SE = 0.21, t = 6.21). The exclusion of 2020 resulted in a notably higher adjusted R^2^ (0.98 vs. 0.85), illustrating the potential distortion effects of the pandemic year on the trend analysis.

Considering the entire age spectrum of 0–19 years, including 2020 in the analysis led to the identification of a breakpoint at 2020 (SE = 0.56), with a pronounced post-breakpoint slope increase (β = 5.40, SE = 1.87, t = 2.88). Conversely, excluding 2020, the breakpoint shifted slightly back to 2017.39 (SE = 0.28) and exhibited a mild increase (β = 2.45, SE = 0.20, t = 12.06). The exclusion of 2020 also resulted in a higher adjusted R^2^ (0.99 vs. 0.93), suggesting a more stable trend analysis over the years without the irregular impact of the pandemic.

### Secondary healthcare– outpatient CAP providers

The average referral rate for initial assessments of CAP at the secondary level of healthcare, covering referrals categorized as very fast, fast, and regular, increased from 4.66 referrals per 1000 individuals aged zero to 17 in the pre-pandemic years of 2018 and 2019 to 5.75 in the period from 2020 to 2022 (Table 1, Appendix 2). This represents a 23% increase. The most pronounced growth was observed in the very fast category, while there was a decline in the regular referral rate. Interestingly, there was no initial decline in 2020 compared to the average referral rate in 2019 and 2020.

**Table 1:** Referral rates for initial assessments with CAP; comparison of the pre-pandemic years 2018–2019 to the period 2020–2022 (for more details, see Appendix 2); data for secondary level of care encompass referrals categorized as very fast, fast, and regular

Year	Urgent referral rate	Very fast referral rate	Fast referral rate	Regular referral rate	Referral rate to secondary CAP
	Per 1000 persons				
2018	0.85	0.74	2.03	1.60	4.37
2019	1.21	1.26	2.08	1.61	4.95
2020	0.96	1.59	1.92	1.14	4.66
2021	1.61	2.69	2.43	1.24	6.36
2022	1.78	2.60	2.56	1.08	6.23
2018–2019	1.03	1.00	2.05	1.61	4.66
2020–2022	1.45	2.29	2.31	1.15	5.75

**Fig. 3:**
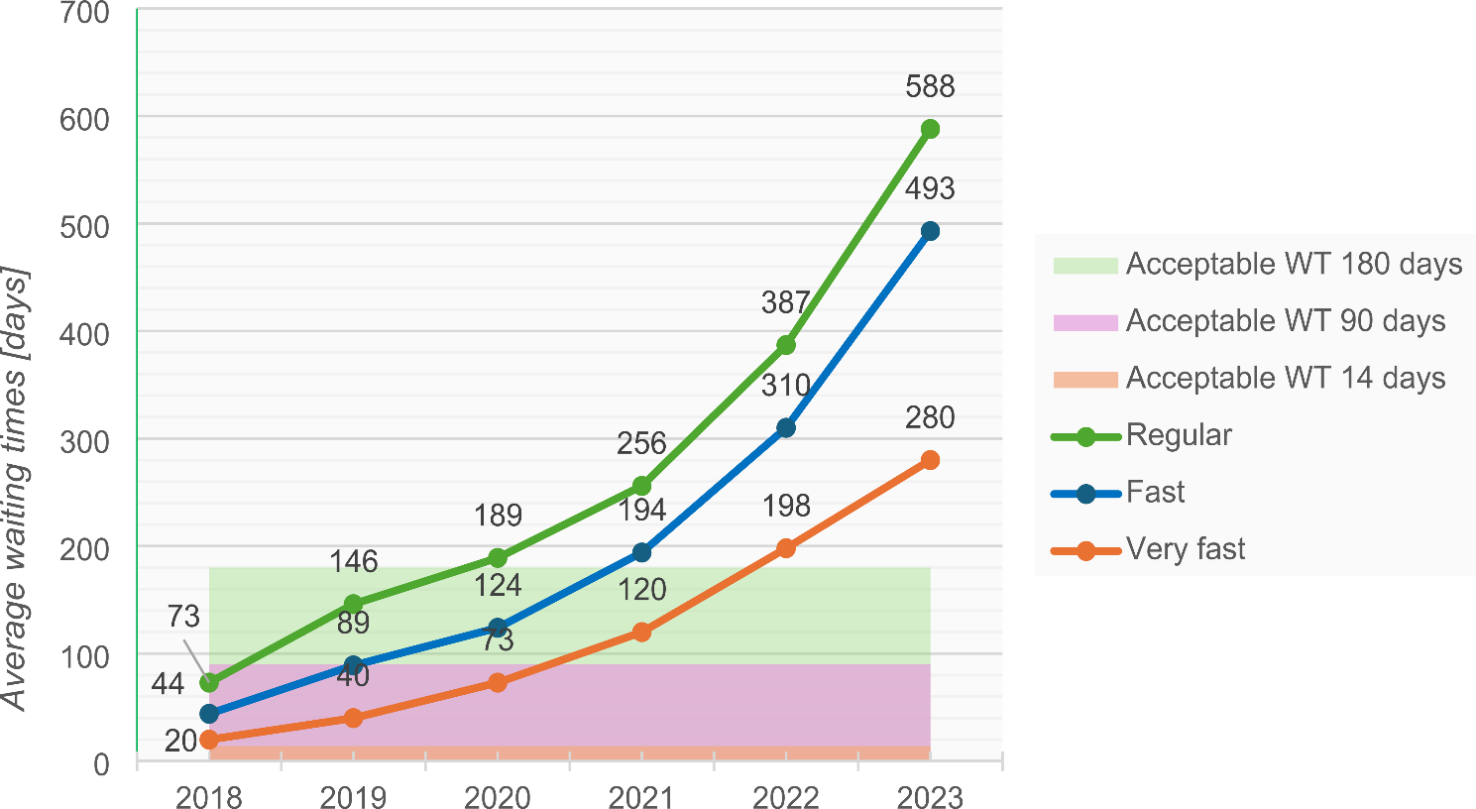
The average waiting times for the initial assessment with a CAP in days from 2018 to 2023. The data are recorded as of 21 August each year. According to the Slovenian Health Insurance Institute, the acceptable waiting times are up to 14 days for a very fast referral, up to three months for a fast referral, and up to six months for a regular referral

The waiting times for various types of referrals have increased significantly since 2018 (see Fig. 3). Of particular concern is the category of very fast referrals, which officially require an initial appointment within 14 days. This benchmark was already slightly exceeded in 2018, but by 2023, the waiting time had extended to an average of 280 days or over 9 months, which exceeds the acceptable waiting time by 2000%. Before the outbreak of the pandemic, waiting times for fast and regular referrals were within the acceptable timeframe. However, in August 2020, the waiting times for all urgency levels exceeded the acceptable thresholds. In August 2023, a child or adolescent in Slovenia had to wait an average of 493 days (16 months) for a fast referral and 588 days (20 months) for a regular referral. The waiting time in August 2023 was four times greater than that in August 2019 for regular referrals and seven times greater than that for very fast referrals.

### Tertiary healthcare– outpatient emergency centres

Table 1 shows a significant increase in the rate of urgent referrals per 1000 persons aged zero to 17 years for an initial assessment with a CAP between 2018 and 2022, with a notable increase of 2.09 times. In 2020, the number of emergencies was lower than that in both the previous year and subsequent years, and there was a 7% decrease in the number of urgent referrals in 2020 compared to the average of 2018 and 2019. Comparing the average referral rate in the pre-pandemic era to the average referral rate from 2020 to 2022, there was a 41% increase in the urgent referral rate.

In Slovenia, all three triage emergency centres are located in the CAP departments of university hospitals. In the interviews, all three department heads emphasized that the vast majority of emergencies require an immediate intervention and rapid follow-up. Therefore, these patients are admitted (to inpatient or outpatient care) until they receive an initial appointment with a secondary CAP provider. As the number of new patients exceeded the number of discharges, all three reported that university outpatient clinics are constantly overloaded. According to their professional judgement, outpatient providers at the secondary level should increase their capacity to prioritize these urgent cases. All three department heads reported that their facilities had not hired additional CAPs, even though the number of urgent patients greatly increased. The operation of these tertiary outpatient clinics relied on the hospital’s existing consultants, who were responsible for urgent outpatient visits in addition to their regular work in their wards and tertiary outpatient clinics. Most emergency assessments were carried out by CAP trainees under the supervision of experienced specialists.

## Discussion

The aim of the present paper was to assess the burden of child and adolescent mental health problems on all levels of the Slovenian mental healthcare system and to compare that data with data from before the COVID-19 pandemic.

After an initial drop in 2020, we observed an increased demand across all three tiers of the Slovenian healthcare system. There was a 20% increase in initial visits for MDR in primary healthcare, a 23% increase in referrals to the secondary level, and a 41% increase in referrals to the tertiary level of care in the period 2020–2022 compared to the pre-pandemic period of 2018–2019. Secondary healthcare reported a four- and sevenfold increase in waiting times for initial assessments for regular and very fast referrals, respectively, from August 2019 to August 2023. The prolonged waiting times indicate a collapse of the outpatient CAP system in Slovenia, leading to an increase in very fast and urgent referrals and, therefore, overwhelming pressure on the 3 triage emergency centres within tertiary hospitals. Due to these extensive waiting times, triage centres staffed by hospital CAP specialists and trainees temporarily accommodated high-risk outpatients, operating perpetually beyond their capacity. Heads of the emergency centres reported a higher proportion of high-risk patients who need immediate treatment.

With the data for the primary healthcare level available from 2008 onwards, we were able to analyse longitudinal trends exclusively for this level of care. From 2008 to 2019, primary healthcare experienced a consistent increase in initial visits for MBD, with an average annual growth rate of 4.5%. The onset of the pandemic resulted in a temporary 18.7% reduction in visits in 2020. However, the subsequent years saw a dramatic surge in visits, yielding an average annual growth rate of 13.9% for the period 2020–2022. Recognizing that growth has been evident since 2008 but has accelerated in recent years, we sought to determine when this acceleration began. Segmented regression analysis revealed that the growth rate has accelerated since 2017 for the age group 0–5, with an extremely small standard error. For the age group 6–14 years, this acceleration began in 2020. Considering all available data, trends for the age group 15–19, as well as for the entire population aged 0–19, began increasing in 2020. Given the pandemic’s effect on people’s behaviour in seeking healthcare, we excluded 2020 data from the models generated by the computer program. This exclusion showed that steeper trends might have actually begun emerging as early as 2017 for the age groups 0–5, 15–19, and 0–19, but not for the 6–14 year-olds.

The reduction in visits and referrals at the beginning of COVID-19 pandemic was attributable to altered healthcare-seeking behaviours, exacerbated by a generally less accessible healthcare system, and was comparable to reports from other countries [8, 19, 26]. A meta-analysis of 42 studies from 18 countries concluded there was a reduction in paediatric emergency visits due to mental health problems; however, the vast majority of included studies compared data only from 2020 with the pre-pandemic year(s), so they probably captured the reduction from the start of the pandemic [8]. A later study observed a 53% initial drop in referrals in the Republic of Ireland in March-May 2020 compared with the same period in 2019. However, in the second half of 2020, referrals and visits due to mental health problems began to grow, culminating in a 180% increase in referrals in November 2020 compared to November 2019 [20]. A Finnish study on the population aged 15–24 years observed a 28% increase in primary healthcare visits due to MBD from 2019 to 2020 and a 102% increase from 2019 to 2021 [27]. In a survey, 84% of 454 Swiss child mental healthcare professionals stated there were more or many more treatment requests and patient registrations in January-March 2021 compared to normal. They also reported on prolonged waiting times [26]. Observations of heads of Slovenian hospital departments for child and adolescent psychiatry regarding the severity of pathology are consistent with other studies [8, 19, 20] and other clinicians’ observations [23]. A meta-analysis by *Madigan et al.* found good evidence for an increase in emergency department visits for attempted suicide during the pandemic– the majority of data applied to the first year of the pandemic (rate ratio 1.22, 90% CI 1.08–1.37) [8].

One explanation for the increasing trends in the use of primary healthcare due to mental health issues could be attributed to changes in the encryption of diagnoses or the expansion of a larger and more accessible network of primary healthcare services. However, neither of these has occurred since 2017. Increasing waiting times at the secondary level could hypothetically result from a reduction in the secondary-level CAP network. Nevertheless, the CAP network has, in fact, expanded, with new CAMH centres for 5–19 year-olds opening after 2020 and functioning within the secondary healthcare system [28]. The developmental outpatient services for children aged 0–5 that function within the primary healthcare system, however, were gradually increased from 21 teams in 2013 to 27 teams in 2022 (Appendix 4). Trends in healthcare indicators across all levels are consistently increasing. Based on primary healthcare data, we can infer that trends were also increasing for secondary and tertiary healthcare before the pandemic. Nevertheless, we cannot definitively determine what the needs would have been like without the pandemic.

A limitation of our study is the absence of secondary- and tertiary-level data prior to 2018. Nonetheless, we included two years pre-pandemic. Another limitation is that one of the three emergency triage centres also accepts patients without a referral [15], probably elevating the actual rate of urgent CAP assessments in Slovenia we didn’t capture in our data. However, the data on their assessments of suicidal children and adolescents also show an initial drop in assessments and hospitalizations at the beginning of 2020 and a gradual increase from the end of 2020 to the summer of 2021. They also show drops in assessments during the school-free months of summer break, which indicates that the pressures of school may be an important factor in the need for emergency child and adolescent psychiatric services [12]. Obtaining data for the year 2023 would greatly aid our analysis; however, despite our efforts, at the time of writing these data were still unavailable from the NIPH. The list of indicators measuring the burden of mental health issues is extensive [29]. For our analysis, we selected one or two indicators for each level of care that appeared most suitable for assessing the burden of MBD among children and adolescents. It is important to consider that this approach, while comprehensive, involves some simplification in interpreting the results.

Our study’s strength lies in the reliability and extensive duration (2008–2022) of the primary healthcare data, encompassing all children and adolescents in Slovenia, as primary healthcare is very accessible for this demographic. This extended timeframe allows for the identification of evolving trends in mental health problems since 2008. While numerous articles have highlighted an increase in MBD and the system overload after the beginning of the pandemic [8, 19, 20, 26, 30, 31], to our knowledge none have contextualised these data within a broader historical perspective. There has also been no similar analytic article on this topic published in Slovenia. Also, other data from the NIPH are very robust, as they encompass all referrals that were issued in Slovenia. Predicted waiting times, which were questioned by politicians in the Slovenian media [32], were confirmed by our cross-sectional study, encompassing 91% of Slovenian CAP outpatient providers, and thus they affirmed the reliability of the official NIPH data on waiting times (Appendix 3).

It would be valuable to compare international longitudinal data about the burden on healthcare systems and differentiate the effects of the pandemic and probable previous influential factors (e.g. social media use, screen time among infants and children, changes in family structures and family burdens, better recognition of mental disorders in children and adolescents, decreasing stigma, etc.). It is also important to continue monitoring the burden on the healthcare system, as the COVID-19 pandemic and its consequences might have a long-term effect on child, adolescent, and adult mental health [10].

In light of our findings, addressing the burgeoning mental health crisis among Slovenia’s young demographic requires a significant and targeted investment of resources in the mental healthcare system, including a targeted investment in human resources with the aim of increasing availability and strengthening the system for the future [30]. However, as it is difficult to find enough professionals to meet the demand with sufficient speed, we advocate for urgent, systemic national action [33, 34]. Slovenia should immediately intensify training in evidence-based programmes for children and families, ideally integrated into the health, social, and education systems. Changes at the national level that promote prevention, such as early attachment work, parent training, limited use of electronic devices, reduction of peer violence, and modernization of the school system to reduce stress and promote emotional and social skills, are urgently needed [18, 23, 30, 31].

## Conclusions

This paper’s main contribution is the comprehensive analysis of the increased burden on Slovenia’s outpatient healthcare system for children and adolescents, highlighting significant trends from 2008 to 2023, and of the profound impacts introduced by the COVID-19 pandemic. By analysing both longitudinal pre-pandemic trends and acute changes during the pandemic, this study shows the escalating healthcare demands within Slovenia, which may reflect a wider trend present in other regions, thus offering valuable insights for global health systems facing similar challenges. The data on service utilization and demand provide important insights into the changing rates of clinically significant mental health problems [18–20, 27]. In the face of new and ongoing crises and the likely long-term concerning trends in the future, continuous monitoring and adaptation of the mental healthcare system is essential for the stability of service provision and the well-being of children and adolescents [11–13]. Addressing the crisis of Slovenian youth requires significant investment in the mental healthcare system, especially in its staff. Urgent national action to promote evidence-based programmes that focus on both treatment and prevention is essential [18, 23, 31, 33].

## Electronic supplementary material

Supplementary Material 1

## Data Availability

No datasets were generated or analysed during the current study.

## Abbreviations

CAMHS: Child and adolescent mental health services
CAP: Child and adolescent psychiatrist
ICD-10: International classification of diseases, 10th revision
MBD: Mental and behavioural disorders
SE: Standard error
SLR: Segmented linear regression analysis
NIPH: National institute of public health
WT: Waiting time
