# Supplementary material for: A “silent storm”: uncovering the escalating crisis in mental healthcare for children and adolescents in Slovenia during and after the COVID-19 pandemic
Source: Child Adolesc Psychiatry Ment Health. 2024 Nov 5;18:140. doi: 10.1186/s13034-024-00811-2 (PMC11536616; doi:10.1186/s13034-024-00811-2)
Supplement: Supplementary file 1 — Supplementary Material 1 [file 13034_2024_811_MOESM1_ESM.docx]

**Appendix 1:**

**1.1 INITIAL visits TO primary health care services due to mental and behavioural disorders for different age groups (2008-2022)** Coloured parts – time during the COVID-19 pandemic. MBD – mental and behavioural disorders. The data include visits to paediatricians, family doctors, child developmental (paediatric) services, and general emergency services.

| **Number of initial visits to primary health care service due to MBD*** | | | | | **Number of inhabitants per age group**** | | | | **Rate of initial visits to primary health care service due to MBD per 1000 persons** | | | | **Annual growth of the rate of first visits at primary healthcare due to MBD** | | | |  |
| --- | --- | --- | --- | --- | --- | --- | --- | --- | --- | --- | --- | --- | --- | --- | --- | --- | --- |
| **YEAR** | **Age 0-5** | **Age 6-14** | **Age 15-19** | **All, 0-19** | **Age 0-5** | **Age 6-14** | **Age 15-19** | **All, 0-19** | **Age 0-5** | **Age 6-14** | **Age 15-19** | **All, 0-19** | **Age 0-5** | **Age 6-14** | **Age 15-19** | **Age 0-19** |  |
| **2008** | 1705 | 2333 | 1933 | 5971 | 113432 | 168879 | 111993 | 394304 | 15.0 | 13.8 | 17.3 | 15.1 |  |  |  |  |  |
| **2009** | 1860 | 2485 | 1863 | 6208 | 118280 | 167229 | 107478 | 392987 | 15.7 | 14.9 | 17.3 | 15.8 | 4.6% | 7.6% | 0.4% | 4.3% |  |
| **2010** | 1712 | 2830 | 1880 | 6422 | 122987 | 166054 | 104301 | 393342 | 13.9 | 17.0 | 18.0 | 16.3 | -0.9% | 3.5% | 4.0% | 3.4% |  |
| **2011** | 1862 | 2704 | 1841 | 6407 | 126806 | 165342 | 100877 | 393025 | 14.7 | 16.4 | 18.2 | 16.3 | -3.4% | -2.6% | 1.2% | -0.2% |  |
| **2012** | 1818 | 2809 | 1838 | 6465 | 130353 | 165180 | 99550 | 395083 | 13.9 | 17.0 | 18.5 | 16.4 | 0.5% | -0.1% | 1.2% | 0.4% |  |
| **2013** | 2012 | 2644 | 1942 | 6598 | 132195 | 167093 | 97464 | 396752 | 15.2 | 15.8 | 19.9 | 16.6 | 1.2% | 6.7% | 7.9% | 1.6% |  |
| **2014** | 2217 | 2875 | 2022 | 7114 | 132218 | 170302 | 95674 | 398194 | 16.8 | 16.9 | 21.1 | 17.9 | 5.7% | -1.7% | 6.1% | 7.4% |  |
| **2015** | 2326 | 3149 | 2030 | 7505 | 130894 | 174325 | 94681 | 399900 | 17.8 | 18.1 | 21.4 | 18.8 | -2.2% | -4.4% | 1.4% | 5.0% |  |
| **2016** | 2196 | 3330 | 2126 | 7652 | 128880 | 178500 | 94096 | 401476 | 17.0 | 18.7 | 22.6 | 19.1 | -3.3% | 3.9% | 5.4% | 1.6% |  |
| **2017** | 2066 | 3386 | 2028 | 7480 | 126743 | 182943 | 92875 | 402561 | 16.3 | 18.5 | 21.8 | 18.6 | -4.0% | -8.3% | -3.4% | -2.5% |  |
| **2018** | 2243 | 3801 | 2374 | 8418 | 124580 | 187251 | 92599 | 404430 | 18.0 | 20.3 | 25.6 | 20.8 | 14.9% | 21.5% | 17.4% | 12.0% |  |
| **2019** | 3327 | 4158 | 2455 | 9940 | 123062 | 191891 | 93255 | 408208 | 27.0 | 21.7 | 26.3 | 24.4 | 4.4% | -12.5% | 2.7% | 17.0% |  |
| **2020** | 2942 | 3273 | 1906 | 8121 | 121160 | 195497 | 93674 | 410331 | 24.3 | 16.7 | 20.3 | 19.8 | -10.2% | -22.7% | -22.7% | -18.7% |  |
| **2021** | 4055 | 4751 | 2934 | 11740 | 119327 | 198454 | 94478 | 412259 | 34.0 | 23.9 | 31.1 | 28.5 | 39.9% | 43.0% | 52.6% | 43.9% |  |
| **2022** | 4809 | 5802 | 3122 | 13733 | 117562 | 199510 | 96890 | 413962 | 40.9 | 29.1 | 32.2 | 33.2 | 20.4% | 21.5% | 3.8% | 16.5% |  |

**1.2 ANNUAL GROWTH RATE OF INITIAL VISITS AT PRIMARY HEALTH CARE SERVICE DUE TO MBD PER 1000 PERSONS AGED 0-19**

The annual growth rate was calculated using the following formula: (Year(X) rate - Year(X-1) rate)/Year(X-1) rate * 100

Average annual growth rate from 2008–2019, age group 0–19: 4.5%

Average annual growth rate from 2020–2022, age group 0–19: 13.9%

**1.3 COMPARISON OF THE RATES OF INITIAL VISITS AT PRIMARY HEALTH CARE SERVICE DUE TO MBD PER 1000 PERSONS AGED 0-19 BETWEEN THE PRE-PANDEMIC YEARS 2018-2019 AND THE PERIOD 2020-2022**

*Sources:*

**National Institute of Public Health (Slovenia). To confirm the data’s authenticity, please contact the Statistical Office of the National Institute of Public Health via email:* [*statisticna.pisarna(at)nijz.si*](mailto:statisticna.pisarna@nijz.si)*.*

***The*Statistical Office of the Republic of Slovenia. *To confirm the data’s authenticity, please contact them via email:* [*gp.surs(at)gov.si*](mailto:gp.surs@gov.si)*.*

| **Year** | **Number of initial visits to primary health care service due to MBD (age 0-19)*** | **Number of inhabitants**  **0-19 years**** | **Rate of initial visits to primary health care service due to MBD per 1000 persons** |
| --- | --- | --- | --- |
| **2018** | 8418 | 404430 | 20.81 |
| **2019** | 9940 | 408208 | 24.35 |
| **2020** | 8121 | 410331 | 19.79 |
| **2021** | 11740 | 412259 | 28.48 |
| **2022** | 13733 | 413962 | 33.17 |
| **2018+2019** | 18358 | 812638 | 22.59 |
| **2020+2021+2022** | 33594 | 1236552 | 27.17 |

**APPENDIX 2**

**2.1 REFERRALS TO CAP, 2008-2022.** ^1^Patients with urgent referrals are assessed in emergency centres at tertiary institutions. ^2^Patients with very fast, fast, and regular referrals are assessed by CAPs at the secondary level.

| **Year** | **Number of urgent referrals*^1^** | **Number of very fast referrals*** | **Number of fast referrals*** | **Number of regular referrals*** | **Number of referrals to secondary CAP*^2^** | **Number of inhabitants**  **0-17 years**** | **Urgent referral rate**  **per 1000 persons*** | **Very fast referral rate**  **per 1000 persons** | **Fast referral rate**  **per 1000 persons** | **Regular referral rate**  **per 1000 persons** | **Referral rate**  **to secondary CAP per 1000 persons**** |
| --- | --- | --- | --- | --- | --- | --- | --- | --- | --- | --- | --- |
| **2018** | 312 | 273 | 745 | 588 | 1606 | 367680 | 0.85 | 0.74 | 2.03 | 1.60 | 4.37 |
| **2019** | 446 | 465 | 769 | 597 | 1831 | 369980 | 1.21 | 1.26 | 2.08 | 1.61 | 4.95 |
| **2020** | 357 | 593 | 716 | 426 | 1735 | 372250 | 0.96 | 1.59 | 1.92 | 1.14 | 4.66 |
| **2021** | 604 | 1007 | 911 | 464 | 2382 | 374260 | 1.61 | 2.69 | 2.43 | 1.24 | 6.36 |
| **2022** | 667 | 975 | 961 | 404 | 2340 | 375524 | 1.78 | 2.60 | 2.56 | 1.08 | 6.23 |
| **2018+2019** | 758 | 738 | 1514 | 1185 | 3437 | 737660 | 1.03 | 1.00 | 2.05 | 1.61 | 4.66 |
| **2020+2021+2022** | 1628 | 2575 | 2588 | 1294 | 6457 | 1122034 | 1.45 | 2.29 | 2.31 | 1.15 | 5.75 |

*Sources:*

**National Institute of Public Health (Slovenia). To confirm the data’s authenticity, please contact the Statistical Office of the National Institute of Public Health via email:* [*statisticna.pisarna(at)nijz.si*](mailto:statisticna.pisarna@nijz.si)*.*

***The*Statistical Office of the Republic of Slovenia. *To confirm the data’s authenticity, please contact them via email:* [*gp.surs(at)gov.si*](mailto:gp.surs@gov.si)*.*

**APPENDIX 3**

**3.1 Secondary healthcare – interviews with outpatient CAP providers**

Because of some doubts in the media about the reliability of the NIPH data on waiting times, we initiated a cross-sectional study to collect data on waiting times directly from secondary-level services. In August 2022, we contacted all 23 outpatient CAP providers in Slovenia, and 21 responded, which corresponds to a response rate of 91%. After introducing the study, we presented a fictitious case of a 16-year-old female high school student who received a very fast referral in an emergency assessment after having attempted suicide. We collected the data available for the first assessment from all the providers.

On average, it took 2.5 phone calls (one to 16 calls) to establish communication. Notably, 3 CAMHCs returned our calls within minutes of an initial unanswered call, while two CAMHCs required the most calls (16 in a single week) and only responded after additional email follow-ups. Despite multiple calls and emails, we were unable to reach one of the outpatient concession clinics. In addition, another outpatient concession clinic declined to participate in our cross-sectional study. Most of the interviewees were people responsible for processing patient referrals, usually registered nurses.

The semi-structured interviews primarily centred on the patient appointment and triage process, and included the following items:

→ Introduction of the researcher and the research, along with a request for collaboration, accompanied by an explanation that their clinic would not be individually named in the article.
→ Do you perform triage or automatically categorize patients based on the urgency level indicated on the referral?
→ Case presentation: a 16-year-old high school student who has completed her first year of high school attempted suicide by ingesting 6 tablets of paracetamol. She was examined at the emergency CAP outpatient clinic. She was not hospitalized; however, she was referred to a CAP with a very fast urgency level. Complicated family dynamics were mentioned in the referral.
→ Based on the data you have, what urgency level would you assign to this fictional case?
→ Please specify the exact date when this adolescent is expected to be seen.

All triage staff confirmed that they performed triage by themselves, and they consistently categorized our hypothetical patient as a very fast-priority case. Eight providers indicated that the case presented would be placed on a special prioritized list due to the suicide attempt, so the typical waiting times for a very fast referral in their triage did not apply to these cases. Two CAP providers were unable to accept new patients due to extended physician absences. The waiting time for the hypothetical patient's first CAP assessment ranged from 1.2 to 13 months, with an average waiting time of 5.5 months.

These interviews were conducted in the second half of August 2022, so the data were then compared with the official NIPH data from August 21, 2022 – the average waiting time in the NIPH records was 6.5 months. Considering that 8 out of 21 CAP providers who participated in our study placed our fictional case on an especially fast waiting list due to her suicide attempt and that the discrepancy from the official NIPH data was only one month, we concluded that the NIPH data on waiting times were accurate.

**3.2 Tertiary healthcare – interviews with heads of CAP hospital departments and triage emergency centres**

In Slovenia, all 3 triage emergency centres are located in the CAP departments of university hospitals.

In August 2022, we also interviewed the heads of all 3 tertiary CAP hospital departments and triage emergency centres, which serve as primary assessment centres for all psychiatric emergencies in the under-18 population. The main objective of these interviews was to gain insight into their strategies for managing the increasing influx of emergency patients.

We presented the following items for the basis of our discussion:
→ Introduction of the researcher and the research, along with a request for collaboration, accompanied by an explanation that their clinic would not be individually named in the article.
→ What are your observations regarding the influx of patients in triage emergency centres after 2020 and the severity of their clinical presentation?
→ What strategies do you employ for the management of these urgent patients?

In the interviews, all 3 department heads emphasized that the vast majority of emergencies require immediate intervention and rapid follow-up. Therefore, these patients are admitted (to inpatient or outpatient care) until they receive an initial appointment with a secondary CAP provider. As the number of new patients exceeded the number of discharges, all 3 reported that university outpatient clinics are constantly overloaded. According to their professional judgement, outpatient providers at the secondary level should increase the capacity to prioritize these urgent cases. All 3 department heads reported that their facilities had not hired additional CAPs, even though the number of urgent patients had greatly increased. These tertiary outpatient clinics relied on the hospital's existing consultants, who were responsible for urgent outpatient visits in addition to their regular work in the wards and tertiary outpatient clinics. Most emergency assessments were carried out by CAP trainees under the supervision of experienced specialists.

**APPENDIX 4**

**4.1 NUMBER OF CHILD DEVELOPMENT OUTPATIENT SERVICE TEAMS IN SLOVENIA, 2013-2023***

| **Year** | **Number of teams** |
| --- | --- |
| **2013** | 21.14 |
| **2014** | 21.64 |
| **2015** | 21.64 |
| **2016** | 21.84 |
| **2017** | 22.34 |
| **2018** | 22.94 |
| **2019** | 23.14 |
| **2020** | 23.59 |
| **2021** | 25.46 |
| **2022** | 27.26 |
| **2023** | 27.36 |

*Sources:*

**The Health Insurance Institute of Slovenia. To confirm the authenticity of the data, please contact the corresponding author, who can connect you with the individual at the Health Insurance Institute of Slovenia, responsible for their statistical data.*

Child development outpatient services are primarily dedicated to children aged 0-5 years with early developmental issues. They function within the primary healthcare system.

They employ a paediatric, registered nurse, physiotherapist and a (clinical) psychologist.
